# The pathophysiology associated with primary (idiopathic) frozen shoulder: A systematic review

**DOI:** 10.1186/s12891-016-1190-9

**Published:** 2016-08-15

**Authors:** Victoria Ryan, Hazel Brown, Catherine J. Minns Lowe, Jeremy S. Lewis

**Affiliations:** 1 Central North West London NHS Foundation Trust, London, UK; 2 Royal National Orthopaedic Hospital NHS Trust, London, UK; 3 Department of Allied Health Professions and Midwifery, School of Health and Social Work, University of Hertfordshire, Hatfield, UK; 4 Central London Community Healthcare NHS Trust, London, UK

**Keywords:** Frozen Shoulder, Adhesive capsulitis, Systematic review, Imaging, Histology

## Abstract

**Background:**

Frozen shoulder is a common yet poorly understood musculoskeletal condition, which for many, is associated with substantial and protracted morbidity. Understanding the pathology associated with this condition may help to improve management. To date this has not been presented in a systematic fashion. As such, the aim of this review was to summarise the pathological changes associated with this primary frozen shoulder.

**Methods:**

Databases: Medline, Embase, CINAHL, AMED, BNI and the Cochrane Library, were searched from inception to 2nd May, 2014. To be included participants must not have undergone any prior intervention. Two reviewers independently conducted the; searches, screening, data extraction and assessment of Risk of Bias using the Cochrane Risk of Bias Assessment Tool for non-Randomised Studies of Interventions (ACROBAT-NRSI). Only English language publications reporting findings in humans were included. The findings were summarised in narrative format.

**Results:**

Thirteen observational studies (involving 417 shoulders) were included in the review. Eight studies reported magnetic resonance imaging or arthrography findings and 5 recorded histological findings. When reported mean ages of the participants ranged from 40.0 to 59.8 years. Duration of symptoms ranged from 0 to 30 months. The majority of studies (*n =* 7) were assessed to be of moderate risk of bias, two studies at high risk and the remaining four were rated as low risk of bias. Study characteristics were poorly reported and there was widespread variety observed between studies in respect of data collection methods and inclusion criteria employed. Pathological changes in the anterior shoulder joint capsule and related structures were commonly reported. Imaging identified pathological changes occurring in the coracohumeral ligament, axillary fold and rotator interval. Obliteration of the subcoracoid fat triangle also appeared to be pathognomonic. Histological studies were inconclusive but suggested that immune, inflammatory and fibrotic changes where associated with primary frozen shoulder.

**Conclusions:**

This systematic review presents a summary of what is currently known about the tissue pathophysiology of primary frozen shoulder. Further studies that use standardised inclusion and exclusion criteria and investigate changes in naïve tissue at different stages of the condition are required.

## Background

Although frozen shoulder is considered to be a common musculoskeletal condition, with reviews reporting up to 5.3 % of the population being affected [1], definitive prevalence and incidence rates remain unknown [2]. The condition is associated with; (often severe) pain, sleep deprivation, anxiety, and disability that may be hugely disruptive and impacts on nearly every aspect of daily living [3]. The average duration of the condition is 30.1 months (range 1 to 3.5 years) [4] but it may be substantially longer [5, 6], and the burden placed upon individuals and health care services may therefore be considered substantial [7].

The term “frozen shoulder” was introduced in 1934 by Codman who described the disorder as “difficult to define, difficult to treat and difficult to explain” [8]; and in many respects this remains true today. Frozen Shoulder (FS) has been classified into primary and secondary conditions [9]. Primary FS (PFS) is characterised by an insidious onset of idiopathic origin whereas secondary FS is associated with a defined event, such as a known intrinsic (such as rotator cuff disease) or extrinsic (such as trauma) cause [10]. FS associated with medical conditions such as diabetes and thyroid disorders are subcategorised as secondary systemic frozen shoulder [11].

Symptoms associated with frozen shoulder include: localised pain, pain with movement, night pain (rendering the patient unable to sleep on the affected side), marked limitation of active and passive range of movement (particularly external rotation) and normal shoulder radiograph findings [8]. However, the absence of definitive diagnostic criteria imposes challenges for clinical diagnosis and management and research [12]. This diagnostic challenge is further complicated by the clinical overlap in signs and symptoms between frozen shoulder and other conditions, such as; rotator cuff tendinopathy, calcific tendonitis or early glenohumeral arthrosis [13, 14]. A recent narrative review suggested thickening of the coracohumeral ligament (CHL), joint capsule and synovium to be diagnostic features for frozen shoulder [15]. However no systematic review has yet collated the data from imaging studies to specify the intra and peri-articular changes that are associated with the condition.

Historically, the pathology of FS has been attributed to structures such as the subacromial bursa and joint capsule [16, 17]. As arthroscopic and microbiological techniques have advanced other structures have been associated with the pathogenesis of the condition: namely, the rotator interval (RI), long head of biceps (LHB) and the CHL [18]. Contemporary histological analyses have identified the presence of inflammatory markers within the asscoiated tissue [19]. Cytokines, such as Tumour Necrosis Factor (TNF) α, Interleukin (IL) 1 α and β and IL-6 have also been identified [20]. In addition, studies have reported high numbers of fibroblasts and myofibroblasts, suggestive of a fibrotic process [21, 22]. An immunological component has also been linked with frozen shoulder; such as the presence of B-lymphocytes, mast cells and macrophages [23]. Such studies have led to the suggestion that FS may begin as an immunological response which escalates to an inflammatory synovitis, eventually leading to fibrosis of the capsule and that future research should focus on disease [15].

The purpose of this systematic review was to identify and synthesise the available evidence regarding the intra and peri-articular pathophysiology of primary frozen shoulder. A secondary aim was to identify deficits in our knowledge that may inform future research. The review was designed to include studies that had investigated the pathology, physiology, physiopathology, neurophysiology, histology, histocytochemistry, microbiology, immunochemistry or immunohistochemistry of the glenohumeral joint and its related structures in adults diagnosed with primary frozen shoulder.

## Methods

This review is reported in accordance with the PRISMA statement for reporting systematic reviews [24].

### Searches

Databases (Medline, Embase, CINAHL, AMED, BNI and the Cochrane Library) were searched from inception until 2nd May, 2014. Searches were performed independently by two researchers (HB and VR). The search strategy was developed using the Population and Intervention component of the PICO formula (Population, Intervention, Comparator and Outcome) [25]. Search terms related to patho-anatomical and pathophysiological changes associated with primary idiopathic frozen shoulder (Table 1). No language restrictions were applied and searches were limited to human studies. In addition to the formal data base searches a reference list search of included publications was also conducted.

**Table 1:** MEDLINE search strategy used in the review

1	SHOULDER JOINT/ (13897)
2	SHOULDER/ (8870)
3	shoulder*.ti,ab. (41413)
4	exp JOINT CAPSULE/ (25623)
5	BURSA, SYNOVIAL/ or CARTILAGE, ARTICULAR/ (23509)
6	LIGAMENTS/ or LIGAMENTS, ARTICULAR/ (17025)
7	subacromial bursa.ti,ab. (207)
8	1 or 2 or 3 or 4 or 5 or 6 or 7 or 8 (107701)
9	ELBOW/ or KNEE/ or HIP/ or ELBOW JOINT/ or exp KNEE JOINT/ or HIP JOINT/ (89002)
10	8 not 9 (92176)
11	JOINT DISEASES/ or CONTRACTURE/ or exp BURSITIS/ (10137)
12	bursit*.ti,ab. (1880)
13	(adhesive and capsul*).ti,ab. (709)
14	(contracted and shoulder*).ti,ab. (79)
15	(stiff and shoulder*).ti,ab. (220)
16	(restricted and shoulder*).ti,ab. (443)
17	((“50” or fifty) and year and old and shoulder*).ti,ab. (142)
18	contracture*.ti,ab. (15710)
19	(capsular and adhes*).ti,ab. (533)
20	ARTHRALGIA/ (4808)
21	SHOULDER PAIN/ (2817)
22	PERIARTHRITIS/ (1087)
23	(frozen and shoulder*).ti,ab. (862)
24	11 or 12 or 13 or 14 or 15 or 16 or 17 or 18 or 19 or 20 or 21 or 22 or 23 (31479)
25	SHOULDER/pa, ph, pp [Pathology, Physiology, Physiopathology] (2414)
26	SHOULDER JOINT/pa, ph, pp [Pathology, Physiology, Physiopathology] (6206)
27	PHYSIOLOGY/ or NEUROPHYSIOLOGY/ (28421)
28	(pathophysiol* or patho-physiol* or physiopathol* or physio-pathol*).ti,ab. (152283)
29	physiology.ti,ab. (78959)
30	HISTOLOGY/ or HISTOCYTOCHEMISTRY/ (74633)
31	(histol* or histop*).ti,ab. (520480)
32	MICROBIOLOGY/ (5837)
33	microbiolog*.ti,ab. (57683)
34	IMMUNOCHEMISTRY/ (9093)
35	IMMUNOHISTOCHEMISTRY/ (246272)
36	immunohistochem*.ti,ab. (236072)
37	25 or 26 or 27 or 28 or 29 or 30 or 31 or 32 or 33 or 34 or 35 or 36 (1197286)
38	10 and 24 and 37 (1397)
39	limit 38 to humans (1336)

Database: Ovid MEDLINE(R) <1946 to 2nd May 2014>

### Eligibility criteria

Studies were included if the participants were diagnosed as having PFS and had undergone combinations of imaging, histological or biochemical analysis of the glenohumeral joint. Studies were excluded if participants were diagnosed with any form of secondary frozen shoulder, such as diabetes, rotator cuff disease or trauma [12]. To reduce confounding the findings, studies were also excluded if participants had undergone previous interventions directly to the shoulder joint (and were termed non-naïve studies). This was because steroid injections may impact on the structure and biochemistry of the tissue [15, 26]. Furthermore, arthrographic distension and capsular release are designed to disrupt the capsule [27] and manipulation under anaesthetic (MUA) may cause intra-articular damage to multiple structures [28]. Translation services were not available thus non English language studies, identified through the search, were subsequently excluded.

### Selection of studies

Two reviewers (HB and VR) reviewed the articles for eligibility and inclusion with a third reviewer (JL) available in the event of consensus not being achieved. Article titles were used to identify relevant studies. Following this, eligibility was checked and recorded on a checklist designed for the review that incorporated PICO criteria. A data extraction form was developed for the review based upon the University of York, Centre for Reviews and Dissemination (2009) guidance [29].

### Data analyses

Following data extraction, the study characteristics were tabulated and the studies synthesized. The variables synthesized in this review were reported findings from imaging studies of the shoulder joint and its related structures, as well as histological, neural and vascular findings. In addition, studies were assessed and their risk of bias appraised. Whether meta-analyses would be possible or appropriate was considered at this point.

#### Risk of bias

Although not always included in systematic reviews investigating pathophysiological mechanisms it was decided a priori to include an assessment of the risk of bias of the studies included in the current systematic review to enhance the validity of conclusion reached. The choice of a risk of bias tool for the review proved difficult as no one tool was perfectly compatible with this type of review. As the review question did not explore diagnostic accuracy the QUADAS-2 tool to evaluate the risk of bias and applicability of primary diagnostic accuracy studies was not considered appropriate. The ACROBAT-NRSI (A Cochrane Risk Of Bias Assessment Tool for Non-Randomized Studies of Interventions) is used when appraising the risk of bias in non-randomized studies that compares the health effects of at least two interventions. Although the current review explored mechanisms rather than interventions, its domains appeared relevant and appropriate to the review and was chosen for use in the current review [30]. Studies were appraised to be at high, moderate or low risk of bias independently by two reviewers (HB and VR) with a third reviewer available in the event of any non-agreement (JL).

## Results

**Fig. 1:**
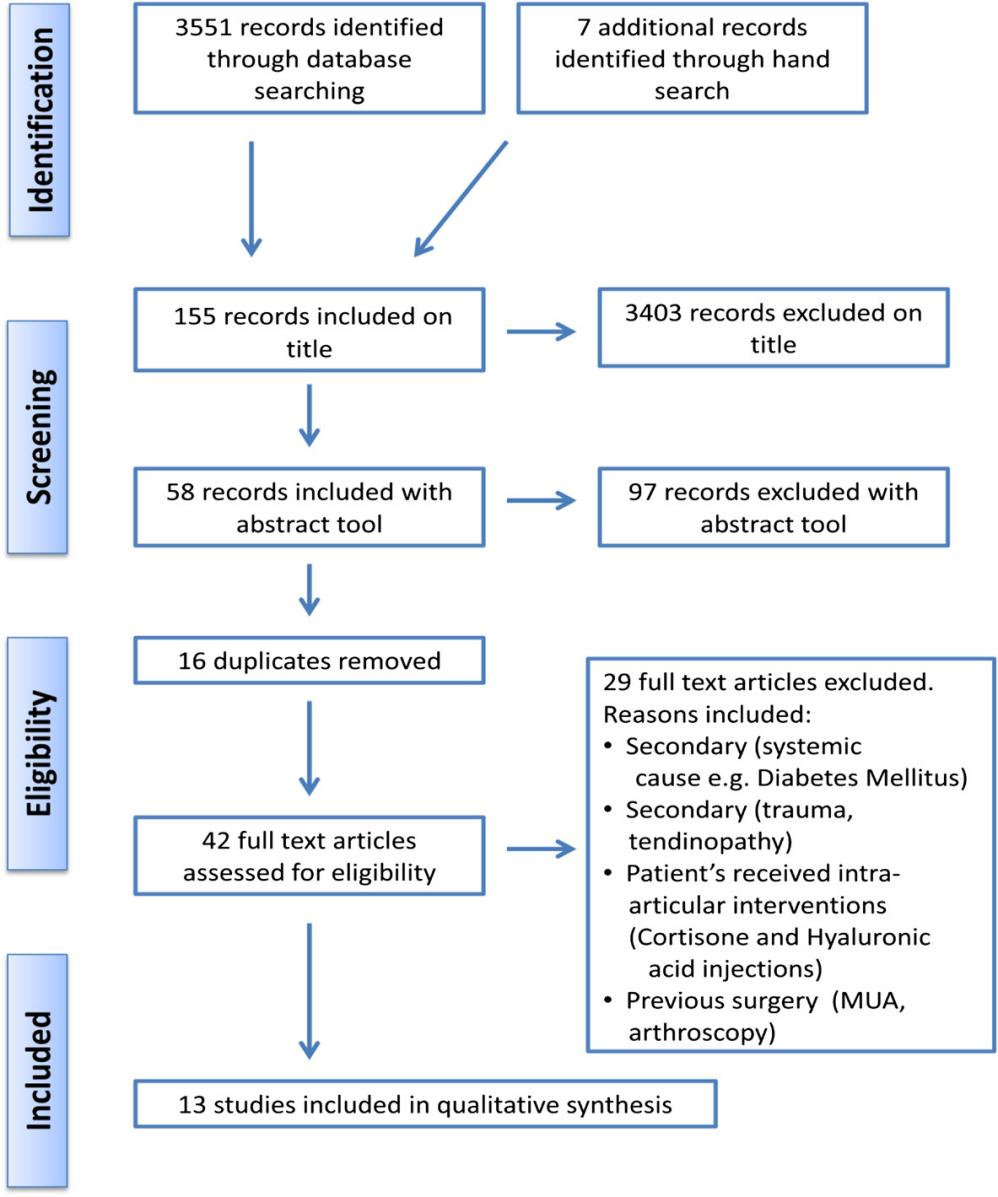
Systematic review protocol

Three thousand five hundred fifty-one potentially relevant studies were identified in searches. Title, abstract and reference list screening identified 58 articles meeting the review criteria. Duplicates (*n =* 16) were removed and the full text of articles read. Thirteen studies met the inclusion criteria for the review and 29 studies were excluded (Table 2). A summary is provided in the PRISMA flow diagram (Fig. 1). The study characteristics are presented in Table 3.

**Table 2:** List of excluded studies

Reference	Secondary					Injection			Surgery	
	Diabetes mellitus	Trauma	Rotator cuff disease	Biceps tendinopathy	Cause not stated	Distension	Corticosteroid injection	Hyaluronic acid injection	MUA	Arthroscopy
Ahn, K., Kang, C., Oh, Y. & Jeong, W. (2012). Correlation between magnetic resonance imaging and clinical impairment in patients with adhesive capsulitis. *Skeletal Radiology. 41*(10),1301-8.	X									
Bunker T. & Anthony. P. (1995). The pathology of frozen shoulder. A Dupuytren-like disease. *Journal of Bone and Joint Surgery*, *77B*(5), 677–683.	X						X			X
Bunker, T., Reilly, J., Baird, K. & Hamblen, D. (2000). Expression of growth factors, cytokines and matrix metalloproteinases in frozen shoulder. *Journal of Bone and Joint Surger,. 82B*(5), 768–773.							X			X
DePalma, A. (1952). Loss of scapulohumeral motion (frozen shoulder). *Annals of Surgery, 135*, 193–204.		X	X	X						
Emig, E., Schweitzer, M., Karasick, D. & Lubowitz, J. (1995). Adhesive capsulitis of the shoulder: MR diagnosis*. American Journal of Roentgenology, 164*(6), 1457–9.										X
Golkalp, G., Algin, O., Yildrim, N. & Yazici, Z. (2011). Adhesive capsulitis: contrast enhanced shoulder MRI findings. *Journal of Medical Imaging and Radiation Oncology, 55*, 119–125.	X									
Gondim Teixeira, P., Balaj, C., Chanson, A., Lecocq, S., Louis, M. & Blum, A. (2012). Adhesive capsulitis of the shoulder: value of inferior glenohumeral ligament signal changes on T2-weighted fat-saturated images*. American Journal of Roentgenology*, *198*(6),589-596.					X		X			
Hagiwara, Y., Ando, A., Onoda, Y., Takemura, T., Minowa, T., Hanagata, N. et al. (2012). Coexistence of fibrotic and chondrogenic process in the capsule of idiopathic frozen shoulders. *Osteoarthritis and Cartilage, 20*, 241–249.	X						X			
Hand, G., Athanasou, N., Matthews, T. & Carr, A. (2007). The pathology of frozen shoulder. *The Journal of Bone and Joint Surgery, 89,* 928–932.	X						X			
Jung, J., Jee, W., Chun, H. Kim, Y., Chung, Y. & Kim, J. (2006). Adhesive capsulitis of the shoulder: evaluation with MR arthrography. *European Radiology, 16*(4), 791–796.										X
Kabbabe, B., Ramkumar, S. & Richardson, M. (2010). Cytogenic analysis of the pathology of frozen shoulder. *International Journal of Shoulder Surgery, 4*(3), 75–78.	X									
Kanbe, K., Inoue, Y. & Chen, Q. (2009). Inducement of mitogen-activated protein kinases in frozen shoulders. *Journal of Orthopaedic Science, 14*, 56–61.								X		
Kanbe, K., Inoue, K. & Inoue, Y. (2008). Dynamic movement of the long head of the biceps tendon in frozen shoulders. *Journal of orthopaedic surgery, 16*(3), 295–299.	X						X	X		
Kim, Y., Kim, J., Lee, Y., Hong, O., Kwon, H. & Ji, J. (2013). Intercellular adhesion molecule-1 (ICAM-1, CD54) is increased in adhesive capsulitis. *The Journal of Bone and Joint Surgery, 95*(4), e18.	X									
Kim, K., Rhee, K. & Shin, H. (2009). Adhesive capsulitis of the shoulder: dimensions of the rotator interval measured with magnetic resonance arthrography. *Journal of Shoulder & Elbow Surgery, 18*(3), 437–42.										X
Lee, M., Ahn, J., Muhle, C., Kim, S., Park, S., Kim, S. et al. (2003). Adhesive capsulitis of the shoulder diagnosis using magnetic resonance arthrography with arthroscopic findings as the standard. *Journal of computer assisted tomography, 27*, 901–906.									X	X
Lee, S., Park, J. & Song, S. (2012). Correlation of MR Arthrographic findings and range of shoulder motions in patients with frozen shoulder. *Musculoskeletal Imaging, 198,* 173-179										X
Lefevre-Colau, M., Drape, J,. Fayad, F., Rannou, F., Diche, T., Minvielle, F. et al. (2005). Magnetic resonance imaging of shoulders with idiopathic adhesive capsulitis: reliability of measures. *European Radiology, 15*(12), 2415–22.							X			
Loew, M., Heichel, T. & Lehner, B. (2005). Intraarticular lesions in primary frozen shoulder after manipulation under general anaesthetic. *Journal of Shoulder and Elbow Surgery, 14*(1), 16–21.	X						X			
Nago, M., Mitsui, Y., Gotoh, M., Nakama, K., Shirachi, I., Higuchi, F. et al. (2010). Hyaluronan modulates cell proliferation and mRNA expression of adhesion-related procollagens and cytokines in glenohumeral synovial/capsular fibroblasts in adhesive capsulitis*. Journal of Orthopaedic Research*, *28*(6), 726–731.			X				X			
Ogilvie-Harris, D., Biggs, D., Fitsialos, D. & MacKay, M. (1995). The resistant frozen shoulder Manipulation verses arthroscopic release. *Clinical orthopaedics and related research, 319,* 238*–*248.						X	X			
Omari, A. & Bunker, T. (2001). Open surgical release for frozen shoulder: Surgical findings and results of the release. *Journal of Shoulder and Elbow Surgery, 10*(4), 353–357.	X									
Ozaki, J., Nakagawa, Y., Sakurai, G. & Tamai, S. (1989). Recalcitrant chronic adhesive capsulitis of the shoulder. Role of contracture of the coracohumeral ligament and rotator interval in pathogenesis and treatment. *Journal of Bone & Joint Surgery - American Volume, 71*(10), 1511–5.		X								
Reeves, B. (1966). Arthrographic changes in frozen and post-traumatic stiff shoulders. *Proceedings of the Royal Society of Medicine, 59*(9), 827–30.		X								
Rodeo, S., Hannafin, J., Tom, J., Warren, R. & Wickiewicz, T. (1997). Immunolocalization of cytokines and their receptors in adhesive capsulitis of the shoulder. *Journal of Orthopaedic Research, 15*(3), 427–436.					X					
Shaikh, A. & Sundaram, M. (2009). Adhesive capsulitis demonstrated on magnetic resonance imaging. *Orthopedics, 32*(1), 61–62.							X			
Tamai, K. & Yamamoto, M. (1997). Abnormal synovium in the frozen shoulder: A preliminary report with dynamic magnetic resonance imaging. *Journal of Shoulder and Elbow Surgery, 6*, 534–543.							X			
Uitvlugt, G., Detrisac, D., Johnson, L., Austin, M. & Johnson, C. (1993). Arthroscopic observations before and after manipulation of frozen shoulder. *Arthroscopy, 9*(2),181-5.		X								
Wiley, A. (1991). Arthroscopic appearance of frozen shoulder. *Arthroscopy*, *7*(2), 138–143.		X								

Articles that were excluded from the study are listed above. The reasons for exclusion are marked in the relevant column

**Table 3:** Characteristics of studies included in the review

Authors, date and country of	Sample size and selection	Inclusion and exclusion criteria	Technique used to gain data	Co-morbidities, previous management, naïve tissue	Findings
Bunker, T. [39] United Kingdom	Sample: *N =* 35. Convenience sample. Gender, age, symptom duration and stage of frozen shoulder not reported Control: Nil	Inclusion:“…fitted the criteria for primary frozen shoulder” Exclusion: Not reported	Arthroscopy + Open release	Co-morbidities, previous management and conservative treatment: Not reported. Tissue extracted from patients who failed to manipulate. Naïve tissue: No	Appearance: Consistent abnormality of the subscapularis bursa. Abnormal villous fronding (large, finely divided expansion) of the synovium. Nodular appearance of the synovium. Histology: Tissue consisted of nodules and laminae of dense collagen (mature' type III). Nodules consisted of a collagen matrix containing fibroblasts arranged alongside layers or bundles of dense collagen. The cell population was moderate to high. Increased vascularity (high or moderate) in seven cases. Immunocytochemistry; Vimentin (a cytocontractile protein) was strongly expressed. Myofibroblasts present. Scanty Leukocytes and macrophages (white blood cells). Synovium: (where present) entirely normal or showed minimal papillary infoldings without increased cell production.
Carbone et al. [31] Italy	Sample: *N =* 50. Convenience sample. Gender not reported. Mean age = 57.9 years (SD = 9) Symptom duration: Greater than 6 weeks. Stage: “In the freezing stage” Control: *N =* 65 RC tear *N =* 50	Inclusion: Painful stiff shoulder (6 weeks), severe pain effecting ADL, specific clinical sign of FS, night pain, painful restriction of active & passive elevation to < 100°& ≥ 50 % restriction of external rotation. Exclusion: age < 40 or > 70 year, wider tear than short-wide RC tear and with subscapularis tear, massive fluid distension of S-A space, concomitant RC tear & FS (full passive ROM), previous treatment/ trauma shoulder girdle/ spine.	MRI	Co morbidities: Not reported Previous management: Patients excluded if they had received treatment for shoulder pain—including oral pain relief. Naive tissue: Yes	Appearance: High intensity signal within the superior subscapularis recess, consistent with fluid distension of the bursa, found in 89.95 % of FS patients. The bursa fluid distension was over, in front of and under the coracoid process.
Carrillon et al. [32] France	Sample: *N =* 25. Convenience sample. M:F = 3:22. Mean age = 51 year Symptom duration: 2–10 months (mean = 6 months. Stage: Not reported Control: RC tear *N =* 15	Inclusion: clinical criteria for FS defined by Codman & Lundberg [9]; Gradually increasing shoulder pain, most severe at rest, ≥ 1 month’s duration, range of anterior elevation of the shoulder no greater than 135°; range of external rotation no >20° and normal GHJ X-ray (no joint space loss, osteophytes, or notches). Exclusion: Not reported.	MRI (Gadolinium enhancement)	Co morbidities and previous management: Not reported. Naive tissue: Unknown	Appearance: MRI: Thickening & postgadolinium enhancement (signs of inflammation) of joint capsule and synovial membrane (*n =* 25), RI (*n =* 25) & axillary recess (*n =* 22). No posterior enhancement (signs of inflammation) noted. Postgadolinium enhancement seen in the subacromial bursa (*n =* 18), supraspinatus & infraspinatus tendons (*n =* 9) and ACJ (*n =* 17). Normal tendons of subscapularis and LHB in all patients (*n =* 25). Arthroscopy (*n =* 2): Major hemorrhagic thickening of the capsule and synovium at the anterior and inferior part of the joint.
Kilian et al. [33] Germany	Sample: *N =* 6. Convenience sample. Gender, mean age, symptom duration not reported. Stage: “Stage II” (Neviaser classification) Control: Shoulder Instability *N =* 6 Dupytrens *N =* 6	Not reported.	Arthroscopy	Co morbidities: Not reported Previous management: Not reported. Naive tissue: Unknown	Histology: Quantitative Reverse Transcription Polymerase Chain Reaction (Q RT-PCR) Used for quantification of DNA sequences: A significant increase (*P <* 0.05) of α1(I) mRNA chains in FS. The quantity of α2(I) mRNA chains between FS, Dupuytren and normal capsular tissue showed no difference. The α1(III) mRNA transcription rate was similar in FS, Dupuytren and normal capsular tissue capsule. Immunohistochemistry: Decreased numbers of fibroblast-like cells with intracellular procollagen I staining recognisable in FS. Weak staining of collagen I in FS and Dupuytren’s tissue when compared to normal capsular tissue. Collagen III staining revealed a corresponding distribution pattern in all 3 groups.
Lho et al. [19] South Korea	Sample: *N =* 14. Convenience sample. Gender, age, symptom duration and stage of frozen shoulder not reported Control: Shoulder Instability *N =* 7	Inclusion: Global restriction shoulder PROM. Arthroscopic confirmation of of hypervascular synovitis& thickened RI &capsule. MRI confirmed no pathology in RI, labrum, LHB or ACJ. Exclusion: Not reported	Arthroscopy	Co morbidities, previous management: Not reported Naive tissue: No	Histology: Elevated IL-1α (Interleukin 1 alpha cytokine) in RI capsule (1.5 +/− 0.15, *P <* 0.05) and SAB (2.3 +/− 0.24, *P <* 0.05), compared to control gp (1.0 +/− 0.01 in joint capsule & 2.0 +/− 0.06 in SAB). Elevated IL-1β (interleukin 1 beta cytokine) in RI capsule only (4.3 +/− 0.3, *P <* 0.05), compared to control gp (3.1 +/− 0.2). Stimulated levels of Tumor necrosis factor alpha cytokine (TNF- α) found in RI capsule (3.1 +/− 0.35, *P <* 0.05) & SAB (3.5 +/− 0.41, *P <* 0.01). Elevated levels of IL-6 (Interleukin 6 cytokine) in SAB only (2.2 +/− 0.3, *P <* 0.01). Cycloogenase COX-1 (enzyme) was increased in the RI capsule only (4.0 +/− 0.14, *P <* 0.05). Cycloogenase COX-2 (enzyme) was increased in the RI capsule (5.0 +/− 0.15, *P <* 0.05) and SAB (6.9 +/− 0 .94, *P <* 0.05) (but not in controls). TNF-α and IL-6 were increased in joint fluid: TNF-α level higher in FS (16.0 +/− 4.04 pg/mL (picograms per millilitre) than controls (10.0 +/− 1.76 pg/mL) (*P <* 0.05). Increased production of IL-6 in FS (21.8 +/− 4.63 pg/mL) compared to controls (3.7 +/− 0.42 pg/mL) (*P <* 0.05).
Li et al. [34] China	Sample: *N =* 72. M:F = 22:50. Convenience sample. Mean age = 53.5 years Symptom duration: 15 weeks—18 months (mean = 9.1 months). Stage: Not reported. Control: *N =* 120	Inclusion: “Clinical evidence of FS”. Insidious onset pain & dysfunction. Clinical criteria; increasing pain &stiffness >15 weeks, most severe at rest with restriction of PROM > 30° for 2 or more planes of movement. Exclusion: Previous trauma or shoulder surgery, tumours, RC tear, Calcium deposit on radiography, rheumatoid Arthritis, osteoarthritis, diabetes mellitus, thyroid/heart/ pulmonary/cervical disease, stroke.	MRI	Co morbidities: Excluded. Previous management: All had undergone medical treatment including anti-inflammatory medication, +/−physiotherapy followed up for 24 months. Naive tissue: No	Appearance: Findings in the FS group, but not in control group:1. High-signal intensity soft tissue in the rotator cuff interval. 2. A thickened inferior glenohumeral ligament (axillary recess).3. A low-signal intensity thickened CHL. The CHL was not visualised in 10 out of 120 shoulders in the control group (8.3 %), and 15 out of 72 shoulders in the frozen shoulder group (20.8 %) (*P <* 0.05). The CHL thickness in FS (3.99+/−1.68 mm) was significantly > control group (3.08+/−1.32 mm), (*P <* 0.001).
Manton et al. [35] United States of America	Sample: *N =* 9. M:F = 7:2. Convenience (retrospective) sampling Mean age = 40 year Symptom duration and stage: Not reported Control: Suspected RC or labral pathology *N =* 19	Inclusion: Arthrographic diagnosis of ≥2 of: Joint volume < 10 ml, poor /absent filling of axillary recess of the joint or biceps tendon sheath, irregularity of capsule insertion, pain after injection of <10 ml of contrast material, or extravasation of contrast material prior to injection of 10 ml or more. Exclusion: Not reported	Direct MRA (Intra-articular Gadopentetate Dimeglumine)	Co morbidities: Not reported. Previous management: No distention or anti- inflammatory injection performed before MRI. Naive tissue: No	Appearance: No SD in amount of fluid in the biceps tendon sheath (*P =* 0.45) or the axillary recess (*P =* 0.37) between FS and controls. No corrugation of the synovium in FS, (In controls n = 7). No abnormalities of the rotator interval capsule in FS (In controls *n =* 7). The average thickness of the synovium and capsule at the axillary recess was 4.1 mm (FS) and 5.1 mm (controls) (*P =* 0.11). The mean thickness of the capsule at the humeral head was 3.0 mm (FS) and 4.0 mm (controls) (*P =* 0.07).
Neviaser, J. [40] United States of America	Sample: *N =* 53 Case series (1 case study). Gender, age, symptom duration and stage of frozen shoulder not reported Control: Nil	Not reported.	Arthrography (Radiographic examination) (Intra-articular Diodrast)	Co morbidities and previous management: Not reported Naive tissue: Unknown	Appearance: Thickening and contracture of capsule with resultant decrease injoint capacity and adherence of the reflected fold causing obliteration of the dependent axillary fold. 42/53 patients had decreased joint capacity, obliteration of the axillary fold and frequently a complete/ almost complete absence of the subscapularis bursa. In every case there was In some instances the subscapularis bursa was obliterated and could not be visualised. The biceps sheath was outlined in the majority of pts. Only 18 % of the shoulders with proved FS showed failure of visualisation of the biceps sheath by arthrogram.
Sofka et al. [14] United States of America	Sample: *N =* 47 M:F = 13:33. Convenience sample Mean age = 53 years Symptom duration and clinical staging: Stage 1:(0–3 months) *n =* 8 Stage 2:(3–9 months) *n =* 23 Stage 3:(9–15 months) *n =* 8 Stage 4:(15–24 months) *n =* 8 Control: Nil	Inclusion:“.....either the presumptive clinical diagnosis of FS or MRI findings suggestive of FS”. Exclusion: Not reported	MRI	Co-morbidities and previous management: Not reported Naive tissue: Unknown	Appearance: Thickening of the axillary pouch ranged from 2–13 mm (average = 7 mm). All subjects demonstrated RI scarring, (mild *n =* 16,moderate *n =* 26, severe = *n =* 5). No SD between the degree of scarring between gps. Analysis of signal intensity of the capsule included *n =* 5 with isointensity (the same intensity), 13 with hypointensity, and 29 with hyperintensity relative to the normal signal of shoulder capsule. Capsular and synovial thickening (in the axillary pouch) demonstrated the most correlation with clinical stage of FS with a mean axillary pouch thickness for; stage 2 (7.5 mm), stage 1 (4.1 mm), stage 3 (5.5 mm), and stage 4 (4.1 mm) (*P <* 0.05). No SD for values for stages 1, 3, and 4 when compared to each other. Evaluation of capsular signal was significant (*P =* 0.02), with hyperintense signal correlating with stage 2.
Song et al. [36] Korea	Sample: *N =*35. M:F = 14:21. Convenience sample. Mean age = 50.1 year Symptom duration: At least 4 weeks. Stage: Not reported Control: *N =* 45	Inclusion: Clinical Diagnosis: painful stiff shoulder for ≥ 4 weeks, severe shoulder pain affecting ADL/work, night pain, painful restriction of active and passive elevation to < 100°, 50 % restriction of external rotation, normal radiologic appearance, no secondary causes. Exclusion: RC tear, calcium deposition on radiograph. Bony abnormalities, such as # of clavicle/ greater tuberosity of the humerus and bony Bankart lesion, shoulder surgery, or > than specified ROM.	Indirect MRA (Intra-venous Gadobutrol)	Co-morbidities and previous management: Not reported. Naive tissue: Unknown	Appearance: FS patients had a significantly thicker joint capsule (5.9 +/− 1.7) in the axillary recess and a significantly thicker enhancing portion (6.5 +/− 2.5) of the axillary recess and of the RI (8.3 +/− 3.4) than control gp (4.2 +/− 1.7; 2.1 +/− 3.0; 3.0 +/− 3.6) (*P <* 0.001). 5 pts with FS (14 %) and 7 controls (16 %) had subacromial bursitis (*P =* 1.0). 3 pts with FS (9 %) and 7 controls (16 %) had OA of the ACJ (*P =* 0.5). No glenohumeral joint effusion was observed in 29 of 35 patients with FS (83 %).
Uhthoff & Boileau [41] France	Sample: *N =* 4 . M;F = 0:4. Convenience sample. Mean age = 60 year Symptom duration: 12 months. Stage: Not reported Control: Nil	Not reported	Arthroscopy	Dupuytren’s (*n =* 1) Previous management: Not reported Naive tissue: Unknown	Appearance: Marked synovial reaction of the GHJ. Histology: Little difference in histological findings in synovial tissue & the extracellular matrix of the posterior & anterior structures. Site of biopsy;(1) synovial tissue & capsule from the posterosuperior part of the joint (*n =* 4); (2) synovial tissue and capsule at the RI (*n =* 4); (3) tissue from the CHL (*n =* 4); (4) synovial tissue and capsule from the axillary fold (*n =* 2); and (5) synovial tissue and inferior capsule in contact with the axillary nerve (*n =* 1). Vimentin (a cytocontractile protein) expression in synovial and endothelial cells was similar at the level of the posterosuperior site and the RI. Vimentin was strongly expressed in cells and extracellular matrix of the capsule at the RI, the CHL, and the axillary fold. No expression for vimentin was detected in cells or in the extracellular matrix from posterosuperior capsule specimens. Desmin not expressed in any section. A marked synovial vascular reaction accompanied by formation of villi was found at all sites (intensity varied among different locations). Presence of fibroplasia was evident at all surgically released sites, and areas of spatially nonaligned type III collagen, containing an increased number of fibroblasts, were separated by strands of spatially aligned type I collagen containing the typical fibrocytes in nearly normal numbers. The simultaneous presence of types I and III collagen was similar at all released sites with the exception of the inferior capsule in which little type III collagen was found. Signs of inflammation or perivascular infiltration were not detected in any section.
Xu et al. [37] Australia	Sample: *N =* 8. M:F = 5:3. Sample: Unclear. Mean age = 58 years Symptom duration: 4–9 months (mean = 6.3 months). Stage: Not reported Control: RC pathology *N =* 10	Inclusion: Pain at night and rest. Radiograph = normal. Decreased ROM under anaesthetic. Evidence of synovial fibroblastic proliferation & associated fibrosis on histological examination of biopsy samples. Exclusion: Previous surgery, radiographic signs of shoulder girdle #, Rheumatoid Arthritis, pts with FS & RC tear at same time.	Arthroscopy	Co morbidities and previous management: Not reported. Naive tissue: Unknown	Appearance: Capsular tissue from FS patients was thickened and hyperaemic. Subsynovial hypercellularity was noted, with fibroblastic proliferation and associated variable, focally prominent collagen production and fibrosis. Associated prominent small vascular channels and vascular congestion was seen. [In RC tissue, plump connective tissue cells in a loose fibrous stroma were noted, vascular proliferation was not present, and fibroblastic proliferation with fibrosis was not evident.]. PGP9.5 (a pan-neuronal marker) and GAP43 (a neuronal membrane protein, nerve marker) immunoreactions: The immunoreactivity pattern of distribution of the nerve markers PGP9.5 and GAP43 was similar in capsular tissue from FS and from controls– Both were mainly seen in the subsynovial tissue adjacent to blood vessels. In the FS tissue, PGP9.5 nerve fibres were often observed close to small blood vessels and within the fibroblastic tissue. The expression of PGP9.5 and GAP43 was significantly higher in FS samples (2.8 +/− 0.2 and 2.4 +/− 0.4 per field) than in rotator cuff tear samples (1.6 +/− 0.6 and 1.3 +/− 0.4 per field, *P <* 0.05). CD34 (a blood vessel marker) immunoreactions: CD34 was strongly expressed in the capsular tissue in 6 FS patients (75 %) but in only 1 rotator cuff tear patient (10 %), supporting increased vascularity in the FS samples. Increased subsynovial vascularity and increased numbers of plump fibroblasts were observed in FS compared with RC patients. Vascular proliferation and congestion in the subsynovial fibrous tissue was seen only in FS.P75 (a nerve growth factor (NGF) receptor - neurotrophin receptor) immunoreactions:P75 was expressed in vascular adventitia (the outer most connective tissue) and nerve fibres around blood vessels and was frequently seen in the subsynovial tissue. Although not everywhere, increased expression of P75 was observed in the FS samples compared with RC patients. Moderate to strong staining for P75 antibody was noted in the capsular tissue in 100 % of FS but only in 30 % of RC samples.
Zhao et al. [38] China	Sample: *N =* 60 M:F = 24:36. Sample: Unclear. Mean age = 50.2 years Symptom duration: 15 weeks - 30 months (mean = 12 months) Stage: “Patients were classified into early or late stage” Further details unclear. Control: *N =* 60	Inclusion: Clinically diagnosed with FS, insidious onset of pain and dysfunction. Clinical criteria: increasing pain and stiffness for > 15 weeks, most severe at rest, with restriction of PROM greater than 30° in two or more planes of movement. Exclusion: Previous surgery or trauma. Neurological disorder involving the upper limbs. Clinical history and clinical examination compatible with RC tear. Presence of calcium deposition on radiographic evaluation, Rheumatoid arthritis, Osteoarthritis.	MRI	Co morbidities: Not reported Previous management: Not reported Naive tissue: Unknown	Appearance: FS pts had a significantly thicker CHL (4.21 mm +/− 0.97) than control subjects (2.12 mm +/− 0.84, *P <* 0.001). Mean thickness of the articular capsule at the RC interval > in FS pts (7.20 mm +/− 2.13) than in controls (4.43 mm +/− 1.16, *P <* 0.05). Partial or complete obliteration of the subcoracoid fat triangle (“subcoracoid triangle sign”) was significantly more frequent in FS pts compared with control subjects (partial obliteration, 22 vs. 2 cases (73 % vs. 13 %); complete obliteration, 8 vs. 1 cases (26 % vs. 1.6 %), *P <* 0.001. Synovitis-like abnormalities around the long biceps tendon were also markedly more frequent in patients than in control subjects (18 vs. 2 cases (60 % vs. 6 %), *P <* 0.05. Patients were not significantly different from control subjects with regard to synovitis-like abnormalities at the articular surface of the subscapularis tendon or in the supraspinatus muscle tendon.

*RC* Rotator Cuff, *ADL* Activities of daily living, *yrs* Years, *FS* Frozen shoulder, *pts* Patients, *CHL* Coracohumeral ligament, *#* Fracture, *ROM* range of movement, *GHJ* Glenohumeral joint, *RI* Rotator interval, *OA* Osteoarthritis, *ACJ* Acromioclavicular joint, *MRI* Magnetic resonance imaging, *MRA* Magnetic resonance arthrogram

All 13 included studies were observational in design. Nine studies included a control group [19, 31–38], four did not [14, 39–41]. Of those using a control group, four included patients with rotator cuff pathology [31, 32, 35, 37], three used asymptomatic controls [34, 36, 38] and two studies included patients with shoulder instability [19, 33]. One study included two control groups [31], one with rotator cuff pathology and the other included people without symptoms. Study characteristics were generally poorly reported and there was widespread variation in diagnosis, methods of sample selection, timing of sample selection and presence of confounding variables such as use of oral medications. Eight out of 13 studies (62 %) based their inclusion criteria on the Codman classification [14, 31, 32, 34, 36, 38, 39, 42]. However, it was evident that there were substantial variations in the interpretation of this classification (Table 3).

The risk of bias data is presented in Table 4. The majority of studies (*n =* 7) were identified as having a moderate risk of bias, with two studies assessed of being at high risk of bias and the remaining four rated as low risk of bias. In general, sample sizes were small, ranging from one to seventy two (average = 28) participants. All studies used convenience sampling. Despite eleven studies identifying potential confounding factors, only six [14, 31, 33–35, 38] reported how they had taken account of them in their study design and/or in their analysis. The risk of bias data and widespread variation between studies did not permit meta-analyses.

**Table 4:** Risk of bias results for the studies included in the review

	Bunker [39]	Carbone et al. [31]	Carrillon et al. [32]	Kilian et al. [33]	Lho et al. [19]	Li et al. [34]	Manton et al. [35]	Neviaser [40]	Sofka et al. [14]	Song et al. [36]	Uhthoff & Boileau [41]	Xu et al. [37]	Zhao et al. [38]
1. Did the study address a clearly focused issue?	Yes	Yes	Yes	Yes	Yes	Yes	Yes	Yes	Yes	Yes	Yes	Yes	Yes
2. Did the authors use an appropriate method to answer their question?	Yes Arthroscopy and Open Release	Yes MRI – No comment on contrast	Yes MRI - Contrast	Yes Arthroscopy	Yes Arthroscopy	Yes MRI – No comment on contrast	Yes Direct MRA	Yes Arthrography	Yes MRI – No Comment On Contrast	Yes Indirect MRI	Yes Arthroscopy	Yes Arthroscopy	Yes MRI– No Comment On Contrast
3. Were the cases recruited in an acceptable way?	No SoC *N =* 35	Yes SoC *N =* 50	Yes SoC *N =* 25	No SoC *N =* 6	No SoC *N =* 17	Yes SoC *N =* 72	No SoC *N =* 9	No SoC *N =* 1	Yes SoC *N =* 47	Yes SoC *N =* 35	No SoC *N =* 4	Can’t Tell *N =* 8	Can’t Tell *N =* 60
4. Were the controls selected in an acceptable way?	No No Control Group	Yes 50 Cuff Tear 65 Control	No No Control Group	Yes 6 Control	Yes 7 Control	Yes 120 controls	Yes 19 Control	No No Control Group	No No Control Group	Yes 45 Control	No No Control Group	Can’t Tell 10 Control	Can’t Tell 60 Control
6. (a) What confounding factors have the authors accounted for?	None Recorded	Gender Age Duration of symptoms Stage of condition Previous Mx	Gender Age Duration of symptoms	Stage of condition	Comorbidities Previous Mx	Gender Age Duration of symptoms Previous Mx Comorbidity	Gender Age Previous Mx	None Recorded	Gender Age Stage of condition Symptom duration	Gender Age	Gender Age Comorbidity Duration of symptoms	Gender Age Comorbidity Duration Of Symptoms	Gender Age Comorbidity Ethnicity Duration of Symptoms
(b) Have the authors taken account of the potential confounding factors in the design and/or in their analysis?	No	Yes Age Comparable Groups - Fs & Rc Tear	No	Yes Stage of condition and Sample	No	Yes Gender affect	Yes Comorbidity Different treatment of Control Group/ “Normals”	No	Yes	No	No	No	Yes
7. Can the results be applied to the local population?	Can’t Tell	No Diagnostic Test described awaiting validation	Yes	Can’t Tell	No	Yes	No	No	Yes	Can’t Tell	No	Can’t Tell	Yes
8. Do the results of this study fit with other available evidence?	Can’t Tell	Yes	Yes	No	Yes	Yes	No	Can’t Tell	Yes	Yes	Yes	Can’t Tell	Yes
Overall risk of bias	High	Low	Moderate	Moderate	Moderate	Low	Mod	High	Low	Mod	Moderate	Moderate	Low

*Mx* management, *SoC* Sample of Convenience, *MRI* Magnetic Resonance Imaging)

### Imaging findings

Magnetic resonance imaging (MRI) findings were reported in five studies [14, 31, 32, 34, 38], with one study using Gadolinium enhancement [32] (Table 3). In descending order of frequency, findings included: a substantially thickened CHL [31, 34, 38]; thickening of the joint capsule in the RI [32, 38] and axillary recess [14, 32]; thickening of the synovial membrane in the RI [32] and axillary recess [14, 32]; partial or complete obliteration of the subcoracoid fat triangle [34, 38]; scarring and or thickening of the RI [14, 38]; fluid distension of the bursa within the superior subscapularis recess [31] and synovitis abnormalities around the LHB tendon [38].

Three studies used contrast enhancement arthrography, with two utilising magnetic resonance angiogram (MRA) [35, 36], and the third, radiology [40]. Arthrography findings were contradictory (Table 3). Song et al. [36] reported substantial thickening of the joint capsule in the axillary recess and the RI. Neviaser [40] reported reduced joint capacity secondary to thickening and contracture of the capsule (region unspecified), obliteration of the axillary fold and often complete or near complete abolition of the subscapularis bursa. In contrast, Manton et al. [35] reported a trend for greater capsular thickness in the axillary recess and at the humeral head and increased synovial thickness in the axillary recess in controls, when compared to patients with FS. They also reported that RI abnormalities were more common in control participants, concluding that there are no useful MRA signs of FS.

### Histology findings

Extensive histological findings were reported (Table 3). Tissue samples demonstrated the following: a dense collagen matrix and high population of fibroblasts and contractile myofibroblasts [19, 21, 33, 41]; a fibrotic process limited to the anterior part of the capsule [41]; elevated levels of inflammatory cytokines in the SAB and anterior capsule [19] and the presence of mature and regenerating nerve fibres in the anterior capsule [37].

Five studies explored the histological and molecular changes associated with idiopathic FS (Table 5). When the study characteristics were reviewed limitations were evident. As previously identified, symptomology, demographics and the stage of the condition were poorly recorded. Secondly, there was substantial diversity between studies with regards to what was being measured. Furthermore, the techniques used to obtain the data also varied (Table 3).

**Table 5:** Inter-operative observations and histological findings

		Bunker [39] Arthroscopy +/− open release *N =* 35	Uhthoff and Boileau [41] Arthroscopy *N =* 4	Xu et al. [37] Arthroscopy *N =* 8
Rotator interval	Appearance	Nodular thickening	No signs of inflammation	----
	Histology	↑ Fibroplasia ↑ Cellularity ↑ Vascularity	↑ Fibroplasia	----
Coraco-humeral ligament	Appearance	----	No signs of inflammation	----
	Histology	----	↑ Fibroplasia ↑ Vascularity	----
Inferior glenohumeral ligament	Appearance	----	No signs of inflammation	----
	Histology	----	----	----
Joint capsule	Appearance	Fibrous contracture in RI area	*Posterosuperior :* No signs of inflammation *Inferior:* No signs of inflammation	*Above subscapularis tendon:* Thickened
	Histology	↑ Vascularity	↑ Fibroplasia ↑ Vascularity	↑ Fibroplasia ↑ Vascularity Neoangiogenesis
Synovium	Appearance	*Between subscapularis bursa and RI:* 4/35 Scarred.	*RI:* Villous *CHL:* No villi *Posterosuperior:* Very villous *Inferior:* No villi *AF:* Very villous	----
	Histology	31/35 Abnormal villous fronding. 31/35 ↑ Vascularity	*RI:* ↑ Vascularity *Posterosuperior:* ↑ Vascularity *AF:* ↑ Vascularity	----
Subscapularis bursa	Appearance	“Consistent abnormalities”	----	----
	Histology	----	----	----
Axillary fold	Appearance	----	No signs of inflammation.	----
	Histology	----	↑ Vascularity	----

N (sample size), ↑ (increased), ↓ (decreased) CHL (coracohumeral ligament), RI (rotator interval), AF (axillary fold), −---(no findings or observations recorded)

### Neuronal and vascular findings

Xu et al. [37] investigated neuronal changes within the condition. They reported elevated levels of several immunoreactive neuronal proteins (GAP43, PGP9.5 and P75) in the anterosuperior joint capsule. The distribution of these proteins was either close to small blood vessels or within fibroblastic tissue. Increased vascularity was a common feature identified in the histology studies; particularly located in the anterosuperior structures but absent in the inferior structures (with the exception of the AF).

## Discussion

### Summary of main findings

This review identified that the anterior shoulder structures in primary frozen shoulder were the location of greatest pathological change and in the subsequent clinical features of the disease, namely a loss of external rotation of the shoulder. The limited number of studies conducting histological analyses did not permit definitive conclusions pertaining to histological changes associated with PFS, however, and in line with previously published research, immune, inflammatory and fibrosis appear to play a role in the pathological process. The extent to which each component contributes and the variance associated with this cannot as yet be determined.

### Clinical inclusion criteria

The review identified substantial variations in interpretation of the Codman classification. Future research must clearly detail defined and standardised diagnostic guidelines, to allow for more accurate and definitive comparisons between findings in studies.

### Imaging

Imaging investigations varied substantially across the included trials and are a potential reason for the variations in findings. Three studies used arthrography, with two using direct arthrography, where contrast material was injected directly into the joint [35, 40]. The basis for this is to permit a more precise visualisation of the intra-articular structures [43]. The contrast material was injected until the capsule distended which occurred at approximately 12–14 ml of fluid [44]. Neviaser [17] reported that normal shoulder joint capacity is between 28–35 ml, often reducing to 5–10 ml in cases of FS. Manton et al. [35] reported a tolerance of less than 10 ml in all nine people with FS. Although the significance of reduced joint capacity in the diagnosis of FS remains uncertain [18, 45–47], the effect of capsular distension when introducing the contrast material may have confounded the published findings relating to the capsule and synovium [35, 47]. Song et al. [36] utilised indirect MRA, where contrast was injected intravenously into an antecubital vein. Indirect MRA requires exercising the joint for 10 to 15 min pre-imaging to increase vascular perfusion to improve flow into the joint [48], and again the influence of this activity on the reported findings is unknown.

There is no definitive guidance as to which imaging modality demonstrates greater diagnostic value in FS, and the heterogeneity of techniques used, and their associated potential confounding factors, limits deriving definitive conclusions relating to the articular and peri-articular changes associated with FS.

### Histology

Symptomology, demographics and disease stage were poorly reported in the studies included in this review. The widespread diversity between studies with regards to what was being measured and how data was collected made comparison and synthesis of findings difficult. The main findings with respect to pathology identified in this review are presented in Tables 5 and 6 and are summarised below.

**Table 6:** Molecular findings

		Bunker [39]	Kilian et al. [33]	Lho et al. [19]	Uhthoff and Boileau [41]	Xu et al. [37]
Techniques used	IHC		X	X	X	X
	ICC	X				
	RTPCR		X	X		
	ELISA			X		
Matrix components	Fibroblasts	↑	↓		↑	
	Myofibroblasts	↑	↓			
Cytokines	IL- 1α			↑		
	IL-1β			↑		
	IL-6			↑		
	TNF-α			↑		
Immune factors	Leukocytes	↓				
	Macrophages	↓				
Neuronal factors	PGP9.5					↑
	GAP43					↑
	P75					↑
Vascular factors	CD34					↑
Enzymes	COX1			↑		
	COX2			↑		

↑ (increased), ↓ (decreased), IHC (immunohistochemistry analysis), ICC (immunocytochemical examination), RTPCR (real time reverse transcription-polymerase chain reaction), ELISA (enzyme-linked immunosorbent assay), IL-1α (interleukin 1 alpha), IL-1β (interleukin 1 beta), IL-6 (interleukin 6), TNF-α (tumour necrosis factor alpha), PGP9.5 (polyclonal rabbit antiprotein gene product 9.5), GAP43 (monoclonal mouse antigrowth-associated protein 43), P75 (nerve growth factor receptor p75), CD34 (monoclonal mouse antihuman CD34), COX1 (cyclooxygenase 1), COX 2 (cyclooxygenase 2)

#### Fibrosis and contracture

Bunker [39] and Uhthoff and Boileau [41] used immunocytochemistry (ICC) and immunohistochemistry (IHC) to review matrix components. Both reported fibroblastic proliferation in the superior capsule and the RI. This is consistent with the imaging findings and with previous histological studies [49, 50]. Vimentin is a cytocontractile protein and its presence may be assessed during ICC. Bunker [39] reported that vimentin was strongly expressed and confirmed that the cells were fibroblasts. In addition, when exposed to a smooth-muscle actin, many of the fibroblasts displayed a differentiation into a myofibroblastic phenotype. The myofibroblast, or contractile fibroblast, is the pathognomonic cell of contractile scar tissue and is found in Dupuytren’s and the other fibromatoses [51]. Kilian et al. [33] used reverse transcription polymerase chain reaction (RTPCR) to study the mRNA (messenger RNA) transcription rates in the fibrosing stage of FS. They reported decreased levels of fibroblast like cells and α1 (III) chains which was indicative of a low number of myofibroblasts. The differing results may be due to samples being acquired at different stages of disease process; Bunker [39] did not supply information regarding stage of the condition or duration of symptoms since onset so comparison of results is challenging. Discrepancies in data may also relate to the way in which the tissue samples were managed. RTPCR evaluates gene expression through the presence of individual cells types, whereas, ICC indicates which proteins those cells are producing [52]. Although they had a relatively small number of participants (*N =* 4) Uhthoff and Boileau [41] conducted a comprehensive study to determine if fibroplasia affects all structures equally. Samples were taken anteriorly, posteriorly, superiorly and inferiorly around the shoulder joint. All structures demonstrated fibroplasia, however, vimentin was strongly expressed anteriorly but was absent in the posterior capsule, leading the authors to suggest that fibroplasia and contracture may be different processes. Their cohort consisted of 4 female subjects with no information pertaining to stage of the condition.

To reduce confounding variables the direct local introduction of medication into the joint was an exclusion criteria for the current review. All patients had failed conservative management; but no studies specified what this included. Common conservative management strategies for FS include oral analgesics and NSAIDs [53]. Therefore, the systemic effects of oral medications should be considered. Evidence in both bone and tendon literature suggests that ibuprofen reduces tensile strength, collagen fibre organisation and fibroblastic proliferation [54]. Almekinders et al. [55] conducted an in-vitro study of the effects of indomethacin on injured human tendon tissue. They reported diminished levels of fibroblast DNA synthesis in the groups treated with indomethacin compared to control. It is important to acknowledge that levels of reported fibroplasia may have been influenced by pharmaceutical preparations potentially prescribed to treat the symptoms.

#### Inflammation and immune modulation

Cyclooxygenases play an important role in inflammation and the collagen catabolic process within peripheral tissues [53]. Lho et al. [19] used RTPCR and IHC and reported increased expression of COX1 in the endothelial cells and stroma of the joint capsule and increased expression of COX2 in the capsule and subacromial bursa of the FS group. Furthermore, levels of IL-1α, IL-β, IL6 and TNF-α also differed between the capsule, bursa and joint fluid. Interleukin and TNF-α are pro-inflammatory cytokines; released from immune cells such as macrophages [54]. This may imply that high numbers of these cells may be present in the joint [56]. Bunker [39] reported low numbers of macrophages and leukocytes in the RI. This variation may be reflective of differing pathological processes between structures and/or that the biopsies were taken from different stages of disease, and possibly different diagnostic criteria. Neither Bunker [39] nor Lho et al. [19] provided sufficient background data regarding their participants to explore this. Furthermore, no comparable studies were included in this SR which again reveals a gap in the evidence base that is worthy of exploration.

#### Neuronal and vascular factors

Pain is associated with FS [3]. Hand et al. [6] conducted a longitudinal study of 223 patients with frozen shoulder with a mean follow up time of 4.4 years (range 2 to 20 years), with 41 % of the patient’s reporting mild to moderate pain and 6 % reported severe pain. To date, few studies have investigated the causes of pain experienced by patients with FS [57]. In this review, Xu et al. [37] investigated neuronal components associated with PFS, reporting elevated levels of several immunoreactive neuronal proteins (GAP43, PGP9.5 and P75) in the anterosuperior joint capsule, close to small blood vessels or within fibroblastic tissue. These findings confirm the presence of mature and regenerating nerve fibres in the anterosuperior capsule and may explain the severe pain experienced by sufferers of the condition in the early stages (less than six months). Increased vascularity was a common feature identified in the histology studies; particularly located in the anterosuperior structures but absent in the inferior structures (with the exception of the axillary fold (AF)). This is consistent with the literature where hypervascularity and angiogenesis have been reported as potential sources of pain due to their association with neovessels [58, 59]. Xu et al. [37] reported stronger expression of CD34 (a haematopoietic cell marker) in the superior joint capsule of the FS group compared RC tears, as a control population. Limited conclusions may be drawn from this study because of the small sample size (*n =* 8). Ryu et al. [58] investigated FS in a diabetic cohort and reported CD34 to be strongly expressed. However, caution must be taken when extrapolating these results as the patient’s had received corticosteroid injections. A recent study by Okuno et al. [59] reported that arterial embolization of neovessels in the RI provided rapid relief of pain in their FS group. Limited knowledge exists regarding the pain mechanisms involved with FS [60]. This SR has provided some insight into possible causes. This knowledge has great significance for clinicians as pain is often the dominant complaint in patients with FS. The literature has suggested that the condition may manifest differently between individuals. A greater understanding would greatly assist clinicians to effectively manage this symptom in their patients. It is clear further research is required.

### Limitations

It is acknowledged that this systematic review has a number of limitations. These are reviewed in the following section.

#### Searches

Only English language publications were included in this review so the introduction of language bias cannot be ruled out. Studies where an English translation could not be sourced were identified during abstract analysis. One reviewer (VR) identified eighteen studies where the full text English article could not be sourced. The second reviewer (HB) identified six of those eighteen. All eighteen studies were excluded. The evidence surrounding language bias is conflicting [30], and it is not known how these non-English publications may have influenced the findings of the current review.

No date restriction was applied to the studies so that all available studies could be identified and included in this systematic review, believed to be the first of this type, in this condition. MRI was first introduced into healthcare in the 1980’s, and over time image quality has advanced substantially [61]. The implication of this is that the reported imaging findings from the earlier studies [32, 35] may lack the sensitivity of those in later studies [31]. This also may have influenced the findings and contributed to reported discrepancies.

#### Inclusion and exclusion criteria

The aim of the review was to investigate the intra and peri-articular pathophysiology of the glenohumeral joint in people diagnosed with primary idiopathic frozen shoulder. Only studies specifying primary frozen shoulder were included as it was not possible to separate the findings from investigations that included both primary and secondary frozen shoulders. This meant that primary frozen shoulder findings may have been missed by excluding studies that incorporated both. The decision to only include samples from people diagnosed with primary FS hopefully generated more homogenised data.

Direct injection of medication into the joint was an exclusion criteria in this review to reduce the potential confounding influencing this may have had on the findings. However, there may be other sources of confounding which might have affected the findings of this review. All patients included in the review had had failed conservative management but none of the studies specified what this included. Common conservative management strategies for frozen shoulder include oral analgesics and NSAIDs [62] and potentially, the systemic effects of such oral medications may have also influenced findings.

#### Widespread variation

The main limitation of this review relates to the included studies. Variations in diagnosis, methods of sample selection, timing of sampling, and confounding variables such as use of oral medications, all may have influenced the reported findings and the conclusions of this review. Meta-analysis was not considered due to the considerable and widespread variance within the included studies [30].

#### Risk of bias

The majority of studies (*n =* 7) were identified as having a moderate risk of bias, with two studies assessed of being at high risk of bias and the remaining four rated as low risk of bias. Study characteristics were poorly reported. There are three possible concerns for this review. The first is, as previously mentioned, a risk of bias tool specifically for use in pathophysiology reviews was not found, meaning there may be specific domains relevant for this type of review which have not been appraised or discussed. The second is that, with only a minority of studies being assessed as low risk of bias, the findings of this review may contain systematic bias [30]. Meta-analyses were not included in this review: it is accepted that meta-analyses of studies that are at risk of bias may be seriously misleading since meta-analysis may simply compound the errors, thus producing an erroneous results which may be interpreted as having more credibility [30]. The third concern is that the ACROBAT-NRSI tool, as a recent development to meet the need for a tool to assess risk of bias in non-randomised studies, has yet to be widely used or evaluated. Further research on the performance of the tool in the future may influence the findings of this review or enable the findings to be placed more appropriately in context.

## Conclusions

This systematic review is the first review to synthesise imaging and histological studies to examine the pathophysiology associated with primary frozen shoulder. The review highlights the role of the anterior shoulder structures in primary frozen shoulder, but there is a lack of available evidence considered at low risk of bias to inform understanding of the pathophysiology of the primary frozen shoulder condition. Consensus regarding inclusion criteria (and the interpretation of the Codman classification criteria) is first required for future research to promote studies providing comparable findings. Following this, further studies that identify findings at clearly defined stages of the condition are required to improve the understanding of the disease continuum. Improved understanding may then inform management specific to each stage of this painful, disabling common condition so that it is no longer difficult to define, treat or explain.

## Abbreviations

ACROBAT-NRSI, a cochrane risk of bias assessment tool for non-randomized studies of interventions; AF, axillary fold; AMED, allied and complementary medicine database; BNI, British nursing index; CHL, coracohumeral ligament; CINAHL, cumulative index to nursing and allied health literature; FS, frozen shoulder; ICC, immunocytochemistry; IHC, immunohistochemistry; IL, interleukin; LHB, long head of biceps; MRA, magnetic resonance angiogram; MRI, magnetic resonance imaging; mRNA, messenger RNA; MUA, manipulation under anaesthetic; NSAIDs, non-steroidal anti-inflammatory drugs; PFS, primary frozen shoulder; PICO, population, intervention, comparator and outcome; PRISMA, preferred reporting items for systematic reviews and meta-analyses; QUADAS-2, quality assessment of diagnostic accuracy studies; RTPCR, reverse transcription polymerase chain reaction; TNF, tumour necrosis factor
